# Action capability constrains visuo‐motor complexity during planning and performance in on‐sight climbing

**DOI:** 10.1111/sms.13789

**Published:** 2020-08-30

**Authors:** Marlene H. van Knobelsdorff, Nikki G. van Bergen, John van der Kamp, Ludovic Seifert, Dominic Orth

**Affiliations:** ^1^ Faculty of Behavioural and Movement Sciences Vrije Universiteit Amsterdam Amsterdam The Netherlands; ^2^ Amsterdam Movement Sciences Amsterdam The Netherlands; ^3^ Institute of Brain and Behavior Amsterdam The Netherlands; ^4^ CETAPS ‐ EA 3832 Faculty of Sport Sciences University of Rouen Normandy France; ^5^ Swinburne University of Technology Melbourne VIC Australia

**Keywords:** affordance‐based control, constraints, entropy, functional movement variability, gaze analysis, sport climbing

## Abstract

The capability to adapt to changing conditions is crucial for safe and successful performance in physical activities and sports. According to the affordance‐based control perspective, individuals act in such a way as to take into account the limits of their capability to act. However, it is not clear how strength interacts with skill in shaping performer‐environment interactions. We, therefore, determined whether fingertip strength influences patterns of gaze and climbing behavior on new routes of ever‐increasing difficulty. We expected that comparatively weaker climbers would show less complex behavior because of an inability to perceive and act. Stronger climbers would show more complex visuo‐motor behavior because more opportunities for action remain, even as route difficulty increases. For very strong climbers the route would not be challenging enough, and less complex patterns suffice. Twenty climbers, ranging from lower grade to elite level participated. Maximum fingertip strength was obtained. Participants previewed and then climbed two separate 3 m long traverses, gradually decreasing in edge depth. Gaze and hip positions were collected for subsequent computation of gaze transition entropy (during preview) and hip displacement entropy (during climbing). Data revealed statistically significant curvilinear relationships between both fingertip strength and gaze transition entropy, and fingertip strength, and hip displacement entropy. Visuo‐motor complexity is scaled by how close the individual must act relative to boundaries of what the environment affords and does not afford for action given the individual constraints. Future research should examine in greater detail relationships between action capabilities and functional movement variability.

## 1 INTRODUCTION

Climbing is a rapidly growing mainstream sport that presents a natural context to examine how individuals safely and skilfully adapt to new constraints on performance.^1^, ^2^ For example, previous work has identified a range of important individual constraints for predicting performance for surpassing new routes (called “on‐sight” climbing).^1^ Among key individual constraints, it is clear that exceptional levels of fingertip strength are essential for high levels of climbing ability and that this is a physical adaptation highly specific to climbing.^3^ A reason fingertip strength increases with ability in climbing is because one way of increasing route difficulty is to decrease the size of climbing holds — as the available surface area of a hold reduces, the less force that can be applied and the greater the individual's fingertip strength needs to be.^4^ Currently it is unclear, however, whether fingertip strength also constrains adaptive visuo‐motor behavior during on‐sight climbing tasks.^1^ In this paper, we experimentally examine links between fingertip strength and how the individual climber adapts, in terms of their visuo‐motor movement variability, to changing climbing surfaces. Variability is assessed from a functional perspective, where the structural properties of movement variability are assessed (such as by using entropy analyses) to examine how the visuo‐motor system adapts to ever‐changing constraints in the environment.^5^ In doing so, we develop a rational for enhancing functional visuo‐motor movement variability for supporting safer and more effective participation in sports and physical activities by integrating fields of strength and conditioning and perceptual‐motor learning.^5^, ^6^, ^7^, ^8^

Climbing includes periods dedicated to previewing and to climbing a route. Route preview involves visually examining a route from the ground and may be considered as a form of “non‐physical practice” of the route,^9^ where individuals may make physical gestures as they imagine how they will climb.^10^ Previous research using verbal reports has shown that during route preview experienced climbers tend to evaluate holds in terms of their functional properties (eg, how holds can be grasped or what movements are available), whereas less experienced individuals are preoccupied with structural properties of holds (eg, their location, shape, or size).^10^ There is also indirect evidence that the capability to perceive functional properties of holds is mediated by the individual's action capabilities to use holds.^11^ For example, Pezzulo, Barca, Bocconi, and Borghi^11^ showed that experienced and inexperienced climbers were equally capable of remembering the location of holds after a period of memorization as long as the route was climbable for both groups. When that route was climbable by experienced climbers and impossible for inexperienced climbers, the experienced climbers recalled significantly more holds than beginners. Furthermore, when the route was impossible for both groups, the expert group's recall performance returned toward the beginners’ level. This suggests the support of motor simulation in recalling holds, provided that the route lies within the individual's action capability (see also^12^).

According to the affordance‐based control hypothesis, the individual behaves in such a way that takes into account the limits of their action capability.^2^, ^13^, ^14^ This would imply that individuals are particularly sensitive to boundaries of what the environment affords and does not afford for action given their action capabilities (ie, the critical affordance boundary). Dicks, Davids, Button^15^ for example, considered the effect of positioning individuals at the limit of their affordance boundaries in a soccer kicking task. They showed that goal keepers tend to delay the moment at which they begin to dive to save a penalty kick on the basis of their maximum diving speed. Accordingly, when evaluating how holds might be grasped and used, it seems likely that maximum fingertip strength is somehow accounted for during visual search — particularly when the individual is required to act near to their affordance boundaries (ie, when the required strength approaches their maximum strength producing capability).

What is less clear in the affordance‐based control perspective is how skill can interact with the task at hand.^16^ For their performance niche, skilled individuals have the capability to adapt a greater variety of functional actions.^17^ That is, skill is characterized by an increase in degeneracy (a capability to use a greater variety of functionally distinct motor actions for a given motor problem).^6^ Experienced climbers, for example, will have encountered a huge variety of hold types during their climbing practice (eg, smooth surfaces, pockets, small and large edges, etc), and their capability to functionally vary their actions to changes in holds is presumably very important for their performance as the overall difficulty of routes increases.^8^, ^18^ At the same time, however, skilled individuals are typically efficient in their performance^19^ and tend to vary their actions only when required or when it serves a functional purpose with respect to changing demands of constraints.^20^, ^21^

**Figure 1:**
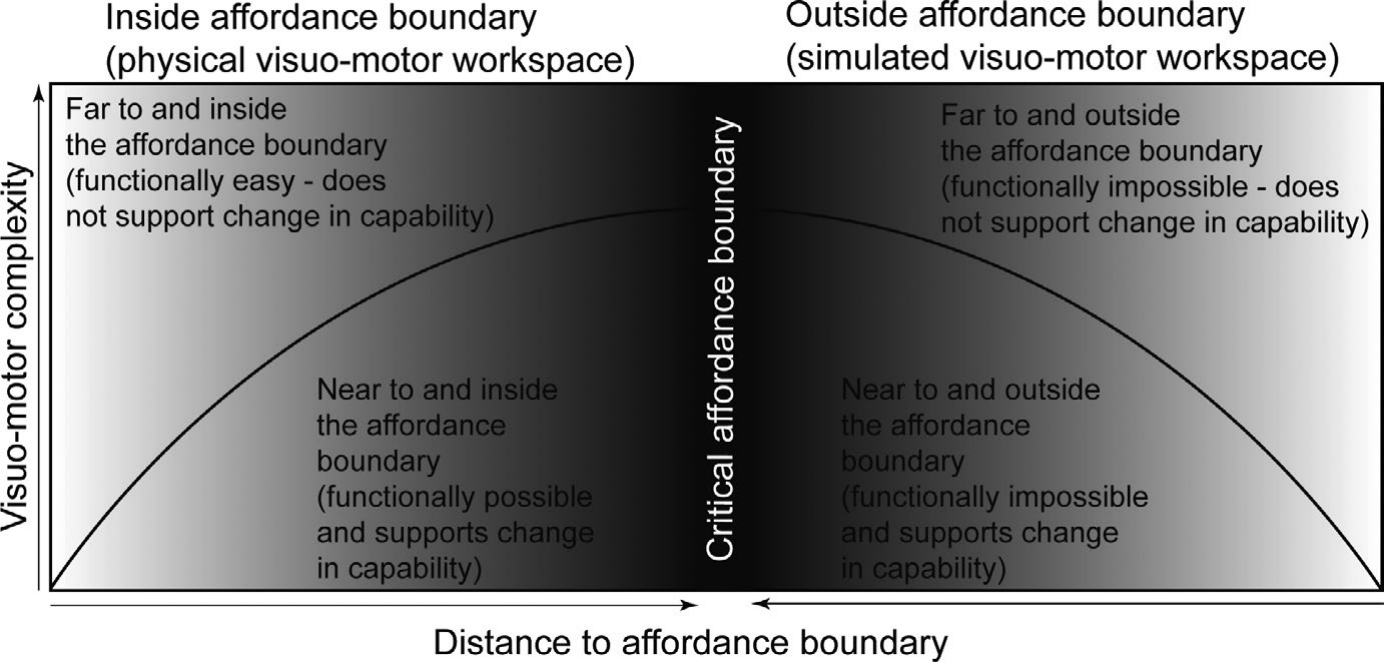
Hypothetical relationship of an individual's expected visuo‐motor complexity when scaling the distance to the boundary separating possible and impossible capability to successfully act

Accounting for expertise in affordance‐based control implies that when holds can be used well within the individual's affordance boundaries (as with a very strong climber on a very easy route), individuals tend to use straightforward (less complex, more predictable) patterns in their visual search during preview and in their movement trajectories during climbing.^22^, ^23^ As holds become more challenging to use with respect to the individual's capabilities (as with a moderately strong climber on a moderately difficult route), the individual may need to explore non‐redundant solutions, such as different ways of grasping or using holds — which would predict an increase in visuo‐motor complexity.^22^, ^23^ Beyond affordance boundaries (such as with a weak climber on a moderately difficult route) the individual is not sensitive to what the route affords for action^10^, ^11^ and hence low complexity in the visuo‐motor pattern would be expected.^24^ The hypothetical relationship between visuo‐motor complexity and proximity to the critical affordance boundary is shown in Figure 1.

The aim of the current study was to determine whether constraints on the action boundary, which in climbing is defined by the fingertip strength relative to hold size,^4^, ^18^ affect visuo‐motor complexity during route preview and climbing under on‐sight conditions. We designed a route that became progressively more challenging so that each climber would at some point be placed at the boundary of their capability to act, and therefore, be required to increase their visuo‐motor complexity to maintain performance. We examined the relationships: (a) between fingertip strength and the complexity of gaze transition patterns using a measure of entropy *during route preview* (referred to as gaze transition entropy^25^), and; (b) between fingertip strength and the complexity of climbing using a measure of the entropy of the movement trajectory at the hip *during climbing* (referred as the geometric index of entropy^26^). Increases in both gaze transition entropy and hip displacement entropy indicate movements are becoming more complicated (or less predictable) and which has been previously associated with increases in functional task difficulty.^22^, ^27^

We hypothesized a non‐linear relationship between fingertip strength and visuo‐motor complexity such that during preview and climbing, complexity of visual search and hip movement would increase close to action boundaries (ie, when the required fingertip strength approaches the maximal fingertip strength) and decrease when far to action boundaries (ie, when the required fingertip strength was inside or outside the maximal fingertip strength).

## 2 METHOD

### 2.1 Participants

Twenty climbers (11 males and 9 females; mean age = 26.5 years, SD = 5.3), ranging in self‐reported redpoint ability level from 9‐25 IRCRA scale^28^ participated. These scores correspond to a range from 5a to 8b (or lower grade to elite) on the French rating scale of difficulty which is well known and used in the European climbing community.^28^ Climbing or bouldering on a regular basis for at least a year was a prerequisite for participation, however, since ability level is correlated with fingertip strength in climbers,^7^ in order to obtain a large degree of variation in fingertip strength we recruited participants practising at all ability levels (see Table 1 for details). The number of participants was based on a power analysis for linear multiple regression (one tailed α = 0.05, effect size *f* ^2^ = 0.4, equivalent to 26% of variance *r* ^2^, β = 0.8, required sample size = 18).^29^ The research project was approved by the institution's ethics committee, the Scientific and Ethical Review Board, Vrije Universiteit Amsterdam, reference: VCWE‐2017‐109, and all participants were involved on the basis of informed consent.

**Table 1:** Descriptive Characteristics of Participants (n = 20)

	Mean	SD
age (y)	26.5	5.3
height (cm)	176.5	9.8
arm span (cm)	176.8	9.5
equipped weight (kg)	69.6	10.4
training years	7.1	5.7
training hours (per wk)	4.8	2.8

Note

n = Sample size.

Abbreviation: SD, Standard deviation.

### 2.2 Experimental design and procedure

**Figure 2:**
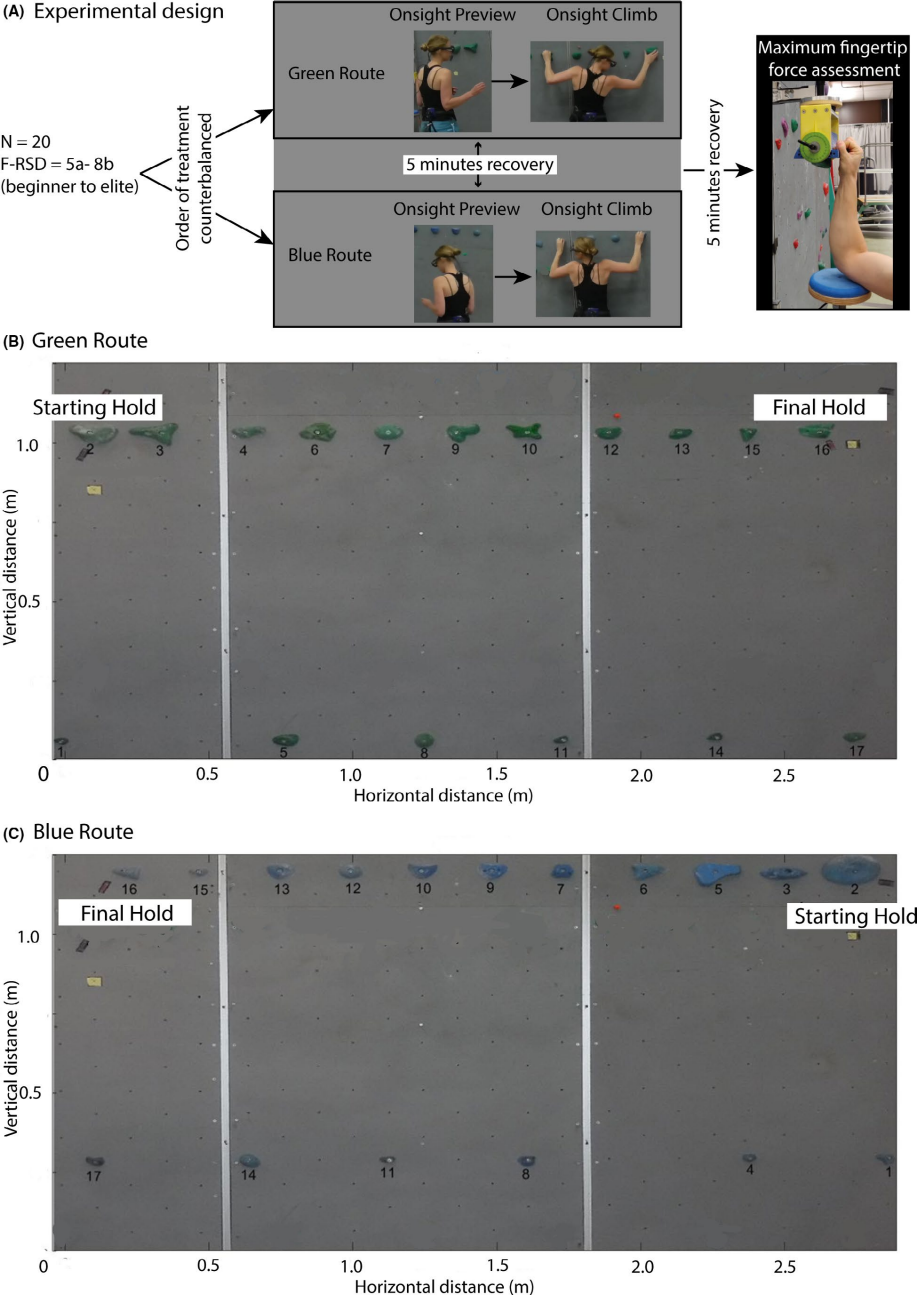
The experimental design (A) and two routes that were previewed and climbed by each participant (B and C)

Participants previewed and then climbed two different 3 meters long traverses (order of treatment was counterbalanced). We changed the direction of each route (one going left to right and one right to left) to ensure effects were not due to direction or handedness, as well as to test replicability. Following the previews and climbs, participants underwent the maximal fingertip strength testing (Figure 2A summarizes the experimental design and Figure 2B,C show each of the routes).

After signing informed consent, information was collected about climbing history and anthropometric measures. Participants then warmed up their hands and feet for 10 minutes using a non‐specific warm‐up. Participants were equipped with the gaze and hip movement registration systems (detailed below). The harness and chalk bag served to carry the equipment. Prior to route preview, participants were equipped with well‐fitting climbing shoes and provided hand chalk as these materials are typically used by climbers and can influence climbing capabilities^30^ and possibly the perception of affordances. Their body weight was then recorded (while fully equipped).

Before data collection, the participants received specific instructions about the preview and climbing task. No explicit instructions were given about climbing techniques and which holds to use, except that the start and end holds required grasping with two hands. Participants were also informed that they had only one opportunity to climb the route. At the start of the preview, the participants saw the route for the first time. The preview consisted of 2‐minute time to look at the route, although participants could stop earlier if they were satisfied. Participants could move around freely in a 2.5 m × 3 m area in front of the wall but they were not allowed to touch the holds. During the route preview the point of gaze relative to the wall plane was recorded (instrumentation described below).

Once the participants were ready, they could immediately start to climb the route. The start of the climb was defined as the moment when both feet had first lost the contact with the floor. The climb finished when both hands grasped the last hold; in case of falling the end of the trial was defined as the last hand contact on a hold before one of the feet touched the ground. During the climb the hip position relative to the wall plane was recorded (described below). The procedure was repeated for each route with a minimum of 5‐minute rest between the end of the climb and the next preview. During the rest period participants sat in an area where they could not see the next route. The short climbing attempts were supposed not to be fatiguing and sufficient rest was enforced after each test to ensure adequate recovery for limited repetition, high‐intensity efforts.^31^

**Figure 3:**
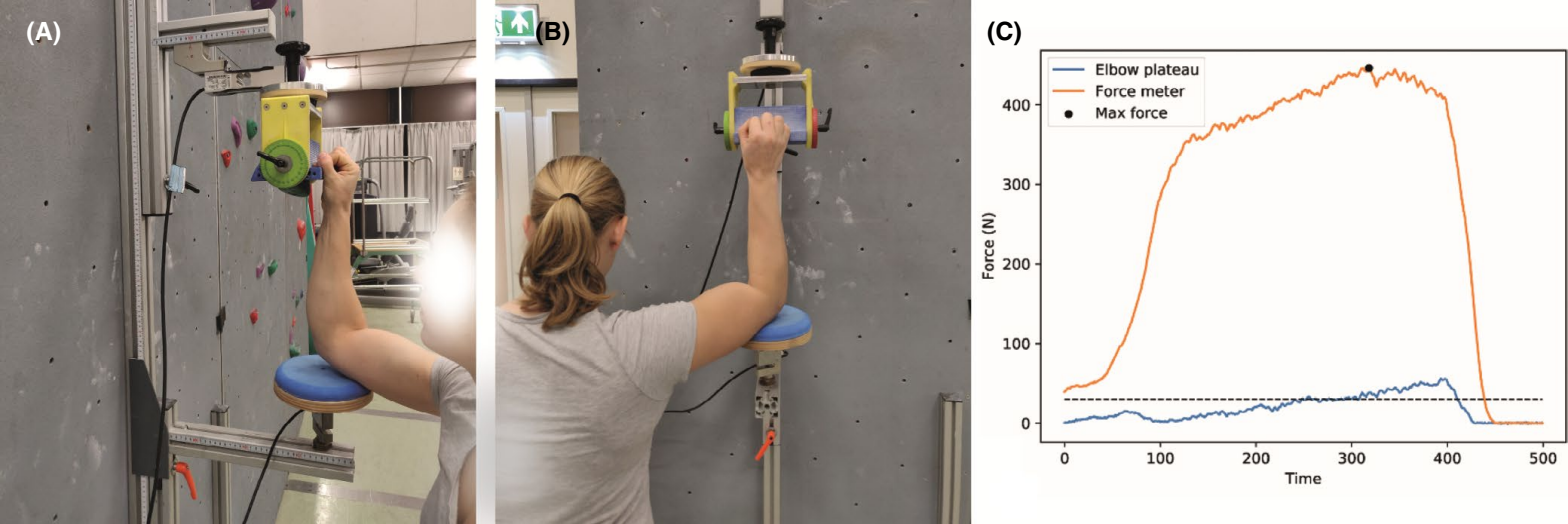
The meta‐grip instrument for assessing maximum voluntary isometric fingertip strength. Panel A shows the DIP test from the side (the edge depth is 1.5 cm). Note the elbow resting height is at acromioclavicular joint height. Panel B shows the elbow position from the back, note the 45 degree offset of the elbow relative to the body. Panel C shows how the maximal isometric force is determined using the peak of the force produced on the instrument (orange line) while also fulfilling the required force applied to the elbow plate (blue line)

After completing the climbing tasks participants were required to have 5‐minute rest before commencing measurement of the maximal fingertip strength. The familiarization protocol consisted of six submaximal contractions with a duration of 10 seconds followed by three seconds of rest, as described by Donath, Wolf.^32^ After the warm‐up, participants received 3 minutes of rest before proceeding to carry out three consecutive maximum voluntary contractions. Each contraction was separated by 30 seconds rest. Participants were verbally encouraged to exert as much force as possible on the strength measurement device. Specifically, participants exerted force only using their fingertips; that is, the distal interphalangeal joint, of their preferred hand against a 1.5 cm edge aligned with the horizontal plane (Figure 3A). The strength measurement took place with the shoulder at 90° anteflexion and 45° abduction, the wrist slightly bended and the elbow at 90° flexion, supported by an adjustable elbow rest (Figure 3B). Participants were instructed to always apply at least 3 kg onto the elbow plate in order to avoid any compensatory behavior and extra involvement of the shoulder. Participants were allowed to use chalk, and the instrument was cleaned with a brush between trials.^32^ The maximum contraction was normalized to the individual's body weight for later analysis.

### 2.3 Instrumentation and data reduction

#### 2.3.1 Routes

Routes were designed so that one route went from left to right (the “Green Route,” Figure 2B), the other from right to left (the “Blue Route,” Figure 2C). Each route was set by a professional route setter and had a 70° inclination relative to the horizontal. Each traverse was composed of 11 hand holds with edges that became increasingly smaller and hence difficult to use and 6 small foot holds (Figure 2B,C).

#### 2.3.2 Measuring preview behavior

Gaze behavior was recorded using SMI^©^ eye tracking glasses (version 2.0, SensoMotoric Instruments Inc). The SMI allows non‐invasive binocular gaze tracking within a range of 80° horizontal and 60° vertical with an accuracy of 0.5°. Data were recorded on a pocket‐sized Samsung Galaxy Note 4 at a sampling rate of 60 Hz. Gaze system calibration took place during the experiment by a three‐point calibration.

As per^23^ fixations (relative stable areas of interest), were detected by a dispersion‐based algorithm. Specifically, fixations were defined as a cluster of successive points within a certain spatial dispersion. The area covered in *x* and *y* coordinates was maximal 100 pixels, and the minimal fixation duration was 90 ms. This is based on the assumption that a shorter gaze duration does not contribute to conscious pickup of information.^33^ Fixation‐based analysis was conducted in the SMI analysis software BeGaze 3.6.52. Fixations recorded on the video were then manually mapped to an image of the wall, corrected for lens distortion (see Figure 2B,C). The final dataset consisting of information about the time, duration, averaged position in pixels, and dispersion of all fixations was further processed in Matlab^®^ R2017a (The MathWorks, Inc). Camera coordinates were converted into world coordinates using a reference grid on the wall. In this study, 17 relevant fixation locations were differentiated for each route, that is, the climbing holds, hereafter to be referred as Areas of Interest (AOIs). Each fixation was assigned an AOI by automatic detection of the nearest hold.

#### 2.3.3 Assessing climbing performance

Climbing behavior was assessed in terms of the geometric index of entropy at the hip during climbing. The Optotrak 3020^®^ motion capture system (Northern Digital Inc), which has an accuracy of 0.5 mm, was used to measure the climber's position relative to the wall during climbing. Two Optotrak cameras were positioned at a 6 meters distance from the climbing wall. A cluster marker consisting of three active infrared markers was secured to a rigid body plane and secured over the climbing harness and positioned on the participant's posterior‐hip at the midline. The position of the cluster marker was recorded as 3D data points at a sampling rate of 120 Hz, and the average of the three markers was used for analysis. Before the experiment, the coordinate system was defined using a calibration cube with the origin set to the bottom left corner of the climbing wall (axes shown in Figure 2). The Optotrak recordings were synchronized to a back camera (Fujifilm FinePix XP80) recording at 60 Hz, using a light triggered simultaneously with the initiation of the Optotrak recording. The start and end points of each climb were then identified for subsequent analysis of the Optotrak data.

#### 2.3.4 Fingertip force assessment

Fingertip force applied onto the meta‐grip instrument was obtained by a single point load cell (Mettler‐Toledo MT1241‐250 kg). A S‐beam load cell (AE sensors STS‐250 kg) was also attached onto the elbow plate, to control for any additional forces originating at the shoulder (recall Figure 3). Both systems sampled at 100 Hz with a National Instruments CompactDAQ system (NI 9218). Before the study commenced, reference weights were used to establish a calibration curve to convert the force output obtained into Newtons. For subsequent analysis, the maximum fingertip force was expressed relative to the individual's body weight.

### 2.4 Dependent variables

To provide an overview of climbers’ ability levels and their outcomes, we computed some traditional dependent variables to summarize behavior at the levels of the gaze displacement and hip displacement. For gaze during preview, these included the number of fixations, average fixation duration, and search rate (the number of fixations divided by the sum of the fixation durations — indicating the number of fixations per unit time). For hip (during climbing) these included distance climbed and success or failure. The primary variables of interest included the maximal fingertip strength (corrected to body weight) for each climber, as well measures of visuo‐motor complexity at the level of gaze displacement during preview (ie, the gaze transition entropy) and at the level of hip displacement during climbing (ie, the geometric index of entropy).

#### 2.4.1 Gaze transition entropy

In order to get insight into the complexity of the visual search behavior visual entropy was calculated by the formula outlined in Ellis, Stark^34^:

$$\text{Gaze}\;\text{Entropy}=\sum_{i=1}^{n}p\left(i\right)\left[\sum_{j=1}^{n}p\left(j/i\right)\log2p\left(j/i\right)\right],i\neq j$$

Firstly, a transition frequency matrix was created, which contained the number of fixations from one AOI (*i*) to the next AOI (*j*). Transitions within an AOI were removed from analysis (*i* ≠ *j*). The probability of fixations on the *i*th AOI, *p(i)* was deduced from the percentage of the total fixation duration on the AOIs. Next, first‐order Markov calculations led to a conditional transition probability matrix, *p(j/i)*. This matrix showed the probability of fixations on the *j*th AOI after fixation on the *i*th AOI. When the fixation location based on the previous fixation location becomes more uncertain (unpredictable), the outcome entropy will be high. In this way the metric construct entropy indicates the degree of redundancy of the gaze behavior — the more redundant (or predictable), the lower the entropy.^25^

#### 2.4.2 Displacement entropy

To obtain the geometric index of entropy (displacement entropy), the 2D coordinates of the convex hull of the climber's trajectory were determined. The convex hull is the global perimeter of the trajectory. The cumulative distance of the climber's trajectory was calculated by summing the differences in position between every two consecutive points. The cumulative distance of the convex hull was calculated in the same way. These two variables are required in order to calculate the displacement entropy (or geometric index of entropy).^26^ Specifically, for a given trajectory *x*, letting ∆*x* be the distance of the path covered by the hip, and ∆*c* the perimeter of the convex hull, displacement entropy is found as:

$$\text{Displacement}\;{\text{Entropy}}_{x}=\frac{\log\left(2*\Delta x\right)‐\log\left(\Delta c\left(x\right)\right)}{\log\left(2\right)}$$

The division by log(2) places the geometric index of entropy in dimensionless terms (bits). For interpretation, we can say that the greater amount of displacement that occurs within a given convex hull, the higher displacement entropy will be and the more complex the movement trajectory is. Conversely, a lower entropy represents a lower climbed distance relative to the convex hull, demonstrating a less complex or more predictable climbing behavior.^26^ Consequently, an individual might only travel 1 m horizontally, but the entropy could be very high. In this case, the path travelled might have a convoluted trajectory or the climber might have gotten “stuck” at some point in the climb and struggled to remain on the wall (in doing so, increasing the total path length of the hip trajectory). Conversely, an individual could travel 3 m horizontally, but the entropy could be very low; in this case, the path travelled might approximate a straight line.

### 2.5 Statistical analysis

The relationships between fingertip strength and gaze transition entropy (during preview), and fingertip strength and displacement entropy (during climbing) were assessed using a two‐step exponential regression between maximal fingertip strength and gaze entropy, and fingertip strength and displacement entropy. Following Cohen^29^ the effect size for power analysis, *r* ^2^ values of 1%‐9% are small, values between 9%‐25% are medium, and values above 25% are large. Power (β) was reported and the threshold set at 0.8. In cases where multiple comparisons are made on the same test, Bonferroni adjustments are made to the reported *P*‐value.

## 3 RESULTS

### 3.1 Route comparisons and global task performance

Table 2 summarizes the outcome measures of interest and provides their simple correlation with fingertip strength. Note that for tests related to hip entropy reduced degrees of freedom are given, because of a marker loss during climbing making computation of displacement entropy impossible (two instances in the blue route and three instances in the green route). Also shown are results of the dependent *t* tests comparing the outcomes for each route. With regards to the *t* tests, there were no significant differences on outcomes between routes. This shows that the outcomes on gaze and hip behavior were generally consistent between routes within individuals, although the green route was slightly easier than the blue route.

**Table 2:** Summary statistics and relationships between outcomes and strength and climbing ability level

Variable	Green route	cor. Strength	cor. IRCRA	Blue route	cor. Strength	cor. IRCRA	Comparisons
	Mean (SD)			Mean (SD)			Dependent *t*
Mean fixation time (s)	0.24 (0.05)	*r*(20) = −0.17, ns	*r*(20) = −0.11, ns	0.26 (0.07)	*r*(20) = −0.05, ns	*r*(20) = −0.09, ns	*t*(19) = 2.12, ns
Fixations (total)	150.7 (148.8)	*r*(20) = 0.03, ns	*r*(20) = 0.10, ns	105.8 (56.3)	*r*(20) = −0.04, ns	*r*(20) = −0.03, ns	*t*(19) = −1.68, ns
Search rate (fixations/s)	4.37 (0.84)	*r*(20) = 0.13, ns	*r*(20) = 0.07, ns	4.09 (0.86)	*r*(20) = 0.08, ns	*r*(20) = 0.14, ns	*t*(19) = −1.78, ns
Gaze transition entropy (bits)	0.79 (0.48)	*r*(20) = 0.13, ns	*r*(20) = 0.21, ns	0.66 (0.31)	*r*(20) = 0.13, ns	*r*(20) = 0.14, ns	*t*(19) = −1.35, ns
Succeeded (total)	7	‐		3	‐		X^2^ = 2.13, ns
Horizontal distance (m)	2.2 (0.4)	*r*(20) = 0.51, *P *= .04*	*r*(20) = 0.55, *P *= .04*	2.1 (0.5)	*r*(20) = 0.51, *P *= .04*	*r*(20) = 0.53, *P *= .04*	*t*(19) = −0.78, ns
Displacement entropy (bits)	1.02 (0.48)	*r*(17) = −0.29, ns	*r*(17) = −0.36, ns	0.94 (0.31)	*r*(18) = −0.11, ns	*r*(20) = −0.16, ns	*t*(16) = −1.05, ns

Note

For all significant findings beta >0.8.

cor. = Pearson's correlation.

* Significant, *P *< .05 (with Bonferroni adjustment).

With regards to the correlation tests, only climbed distance was associated with fingertip strength (stronger climbers climbed further than weaker climbers) and ability level (better climbers climbed further than worse climbers). The non‐significant correlation between strength and ability for behavioral variables was expected because the route difficulty constantly varied and the individuals observed had a broad range in ability and strength.

### 3.2 Preview analysis: Gaze transition entropy outcomes

**Figure 4:**
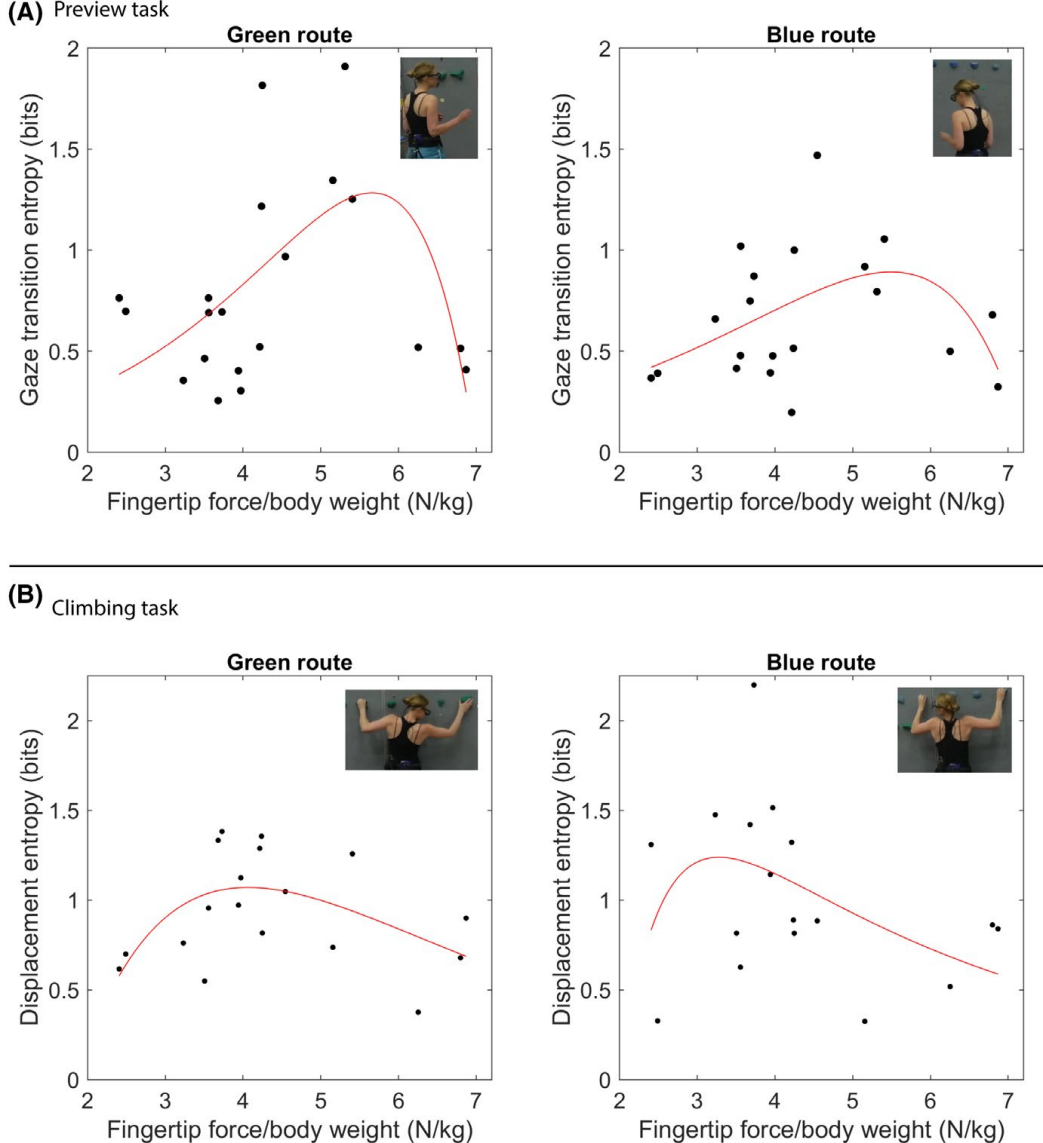
A, Associations between fingertip strength and gaze entropy on the green (left) and blue (right) routes during on‐sight preview. B, Displacement entropy during climbing relative to fingertip strength on the green (left) and blue (right) routes

To assess the relationship between fingertip strength and gaze transition entropy during the route preview task for the blue and green routes, a single group curvilinear regression was carried out.^35^ As expected, for each route the first term exponential regression was not significant for the green route, *r* ^2^ = 0.02, *df* = 18, *P* = 0.56, or the blue route, *r* ^2^ = 0.01, *df* = 18, *P* = 0.56. The two‐term exponential was significant for both the green route, *r* ^2^ = 0.35, *df* = 16, *P* = 0.01, β = 0.95, and blue route, *r* ^2^ = 0.24, *df* = 16, *P* = 0.04, β = 0.91. The increase in r^2^ squared from single to two‐term was also significant for the green route, *P* = 0.003, and the blue route, *P* = 0.02. Figure 4A represents the line fit which shows that for both routes gaze entropy tends to be low with low levels of strength, high with moderate levels of fingertip strength, and low with very high levels of fingertip strength. Ability level and gaze were also tested for a possible curvilinear relationship (which would indicate that ability level is a competing and correlated skill factor that could also explain the visuo‐motor complexity outcome). The two‐term exponential fit was not significant for either the green route, *r* ^2^ = 0.16, *df* = 18, *P* = 0.21, or the blue route, *r* ^2^ = 0.16, *df* = 18, *P* = 0.21.

### 3.3 Climbing analysis: Geometric index of entropy outcomes

For the climbing task for each route, the hip displacement entropy data of the climbers also followed a curvilinear trend with respect to fingertip strength. Specifically, the first term exponential regression was not significant for the green route, *r* ^2^ = 0.01, *df* = 16, *P* = 0.62, or the blue route, *r* ^2^ = 0.01, *df* = 15, *P* = 0.62. The two‐term exponential was significant for the green route, *r* ^2^ = 0.35, *df* = 14, *P* = 0.02, β = 0.95, but was not significant for the blue route, *r* ^2^ = 0.24, *df* = 13, *P* = 0.07. The increase in *r* ^2^ squared from single to two‐term was also significant for the green route, *P* = 0.007, and the blue route, *P* = 0.04. As above, ability level and hip entropy were also tested for a possible curvilinear relationship. Similarly, the two‐term exponential fit was not significant for either the green route, *r* ^2^ = 0.16, *df* = 15, *P* = 0.26, or the blue route, *r* ^2^ = 0.16, *df* = 13, *P* = 0.28.

Figure 4B shows the data line fit for the displacement entropy relative to fingertip strength. These trends indicate an inverted U‐shape where weak climbers tended to have low entropy, the moderately strong climbers had a high entropy and the very strong climbers had a low entropy.

## 4 DISCUSSION

**Figure 5:**
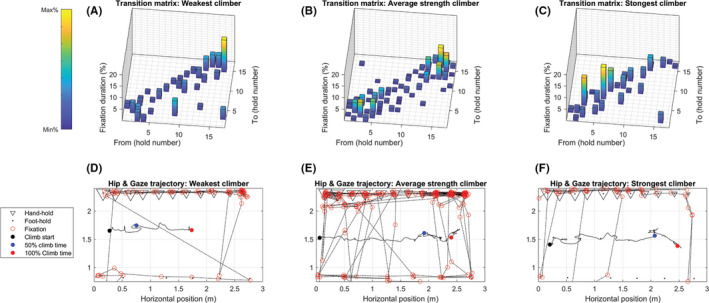
Individual data of the weakest climber (left column), climber closest to average strength (middle column), and the strongest climber (right column) are exemplified. On the top row transitions of fixations between holds during preview are shown. The height of columns shows relative fixation duration, the distribution indicates predictability. The bottom row represents the visual scan path during preview and the hip trajectory during the climb

This study explored the role of individual's action capabilities in affordance‐based control. We expected that acting close to action boundaries would increase the complexity of gaze and climbing behavior whereas acting far from the affordance boundary would reduce complexity (recall Figure 1). The findings indicate that during on‐sight preview and climbing on routes that progressively increase in difficulty: comparatively weak and very strong climbers at the fingertips use less complex visuo‐motor movement patterns and; moderately strong climbers use more complex visuo‐motor movement patterns. This interpretation is expanded on below and is exemplified with individual data coming from three participants (Figure 5). Each participant summarized in Figure 5 is a member of one of the above‐described subgroups. Specifically, we chose the weakest climber, the climber with closest to average strength, and the strongest climber.

The gaze transition entropy indicates that during route preview the weaker climbers visually examined the route, transitioning from one hold to the next, using a redundant, highly repetitive gaze pattern. The transition matrix in Figure 5A exemplifies this showing how gaze transition behavior tended to be narrow, going from one hold to the next in sequence (ie, along the diagonal). The weakest climbers were not able to perceive ways of adapting/changing their action during route preview, and consequently showed a simple visual search patterning. Their general inability to progress through the route (ie, weaker climbers fell significantly earlier than stronger climbers) would suggest that once climbers were examining holds that were beyond their affordance boundaries they either repeated visual search patterns (characterized by an increased redundancy) or they stopped search. It can also be noted that while in Figure 5A the weakest climber spent more time viewing the smaller holds, this did not lead to more complex visual search (it remained simple). The visual plot of the gaze trajectory during preview (Figure 5D) confirms this hold‐by‐hold pattern. The weaker climber looked through the route from the start to the end (here: left to right). (Similar to what Seifert, Cordier, Orth, Courtine, Croft^23^ referred to as an ascending search strategy). The outcome on the climbing task was also consistent with the visual search complexity. The weaker climbers tended to fall early and abruptly, showing little to no capability to adapt to changes in hold sizes, and their low levels of hip displacement entropy reflect this (Figure 5D hip trajectory data exemplifies this).

More complex gaze patterns during preview were found in the moderately strong climbers. They tended to use more diffuse gaze patterns by fixating across holds, subsequently using less repetitive ways of examining holds — suggesting either a more explorative or in‐depth visual search (see Figure 5B, middle transition matrix). The fixations on the transition matrix are more spread out, which reflects a higher entropy. Although, it is not clear to what extent the increase in gaze transition entropy could reflect a tendency to visually explore a greater variety of alternative strategies throughout the entire route or be localized to specific parts of the route.^12^, ^23^ The more complex search pattern of a moderately strong climber is visualized in Figure 5E. A combination of different search patterns seem to emerge, in which a pattern of looking back and forward is alternated with looking from hand to foot holds (Seifert, Cordier, Orth, Courtine, Croft^23^ referred to this as a combination of zigzagging and blocked strategies). During the climbing task, all of the moderately strong climbers also fell. Generally, the stronger the climbers, the more they were able to struggle to maintain contact with the wall, leading to an increase in displacement entropy. This is particularly evident in the second half of the climbed trajectory in Figure 5E, where the displacement becomes increasingly varied/convoluted.

In very strong individuals, the entire route is well within their capability — since they do not need to adapt alternative ways of climbing through the route to be successful, their visuo‐motor complexity would be low. Following this hypothesis, it was found that the three strongest climbers (who also completed the route) showed a low gaze and hip entropy. The behavior of the strongest climbers drove the curvilinear relationships, which would otherwise have been positive linear. Similar to the weakest climbers, the strongest climbers shifted gaze from one hold to the other in a structurally simple and predictable manner (Figure 5C,F), and in contrast to the explorative search of moderately strong climbers. Although the weakest and strongest climbers showed both a straightforward pattern, indicated by the narrow transition matrices, the search pattern of the strongest climber is more functional. Whereas the weakest climber showed a very global search pattern, examining first the hand holds, and then foot holds, the strongest climber examined hand and foot holds during the same scan sequences, resulting in a zigzagging pattern.^23^ Figure 5C also indicates a longer period of time spent transitioning from/to holds at the easy part of the route, possibly indicating a routine starting strategy of the strongest climber (and also reinforcing the importance of considering the structural qualities of visual search). The strongest climbers were also successful in surpassing the route in a very fluid manner with low displacement entropy. Skilled climbers, climbing within their ability level, move in an energetically efficient manner. This can be accomplished by moving the center of mass up and downwards as analogous to an inverted pendulum and thereby increasing the vertical hip variability.^26^, ^36^ In these respects, the hip trajectory in Figure 5F shows the strongest climber could afford to make larger fluent movements at the beginning of the climb and yet also shows a more constrained movement trajectory toward the end of the route. The fluency of the climb is verified by a low entropy (ie, the climbed path relative to convex hull is low). This suggests that the strongest climbers were able to successfully rely on their initial search strategy and were not challenged to adapt a broader range of solutions at each hold since the route did not position them close enough to the limits of their affordance boundaries.

With respect to limitations, it should be noted that visual search strategies are derived from a fixation‐based analysis, which involves some questionable assumptions.^2^ Another limitation of this study concerns the route design. In our aim to examine the role of fingertip strength in visuo‐motor behavior, the route was set in such a way that fingertip strength was the determining factor for route progression. To accomplish this purpose, a traverse was preferred to a vertical climbing route. Additionally, the curvilinear relationships were driven by a relatively small number of climbers, and although we did show these findings were consistent across two different routes, further research is needed to replicate these findings.

So far, a difficult question in the literature to address has been to measure and understand how variation in visual search, particularly under changing constraints, supports performance.^2^ Our data suggest climbers’ action capabilities relative to hold difficulty to use, shape gaze patterns and presumably information pickup. As climbers become attuned to how to use holds relative to their individual capacities, they can benefit from route preview by (more accurately) identifying regions of a route that will present a challenge while climbing. At particularly challenging areas (or as they approach their affordance boundary), it may be useful to the climber to dwell and consider as many (non‐redundant) ways of climbing these areas as possible. Even when close but outside of the affordance boundary, visuo‐motor complexity remains elevated as the individual struggles to successfully adapt movement solutions to the task at hand. From a practical perspective, this also suggests the developing broad repertoire of movement solutions (ie, increase degeneracy) may extend the critical affordance boundary.^6^

This region (close to the affordance boundary) has also been implicated as increasing the learning opportunity for the individual because it stimulates adaptation that is carried over longer time scales.^21^, ^37^ Because some degree of change in how actions are organized for task success, a hallmark feature of behavior around these regions is the tendency for the individual to increase their movement variability.^22^, ^38^, ^39^ Hence, future research might consider how visuo‐motor complexity can be used as an indicator of learning potential for the design of perceptual‐motor tasks (such as in^27^). Another issue is to establish in what ways functional strength supports a greater variety of actions, including in adapting to more complex or difficult movement problems. For instance, does improving an individual's functional strength enhance capabilities to overcome more challenging environmental and task constraints, and if so (which it presumably does), is this because functional strength increases functional movement variability? Also, how might injuries influence visuo‐motor complexity, can changes therein reflect new strategies arising from the changes in affordances that might accompany injury?^40^ Finally, of considerable interest is to understand how visual search complexity during preview might map to motor complexity. For example, it is possible that visual search complexity might indicate the individual's preferred affordance boundary — which may or may not align with the critical boundary. In this case, it could be hypothesized that (c)overt simulation/perception can extend/limit the critical affordance boundary.

## 5 PERSPECTIVE

Findings suggest a curvilinear relationship between action capabilities and gaze *and* climbing complexity such that on the same route: (a) Weaker climbers have more redundant (less complex) visuo‐motor behavior; (b) stronger climbers have less redundant (more complex) visuo‐motor behavior, and; (c) very strong climbers have more redundant visuo‐motor behavior. Variability in gaze behavior can reflect adaptive organization of behavioral strategies relative to the opportunities for action that can be perceived and acted on by the individual. Gaze dynamics associated with preparation to perform a climbing route have a corresponding signature in behavioral dynamics observed in performatory behavior.

In sum, visuo‐motor complexity was positively related with fingertip strength when acting near the individual's performance limits. The findings of the study suggest that climbers account for affordance boundaries when searching for solutions to climbing problems. The complexity of visuo‐motor behavior seems to be influenced by the sensitivity to a changing hold difficulty relative to fingertip strength. Understanding the reason holds are perceived as (un)usable for an individual can facilitate an individualized learning process.

Furthermore, greater interface between strength and conditioning and motor learning can lead to more innovative learning design practices for each discipline. For instance, improving strength across a range of movement patterns to provide athletes with options and thereby more flexibility to explore during visuo‐motor search. This should increase opportunities for affordance perception and hence performance.

Another option may be to determine what training activities can increase the individual's exploratory capabilities at the visuo‐motor level. For example, in climbing, improving active recovery might allow climbers to more effectively exploit visual search opportunities at rest locations. These same principles can be extended to a broad range of sports where exploration has been linked to addressing dynamic motor problems.

## CONFLICT OF INTEREST

The authors have no conflicts of interest to declare.
